# Supplementary material for: Characterization of Three Novel Viruses from the Families Nyamiviridae, Orthomyxoviridae, and Peribunyaviridae, Isolated from Dead Birds Collected during West Nile Virus Surveillance in Harris County, Texas
Source: Viruses. 2019 Oct 10;11(10):927. doi: 10.3390/v11100927 (PMC6832935; doi:10.3390/v11100927)
Supplement: Supplementary file 1 [file viruses-11-00927-s001.pdf]

```
NYMV_L          M----EGPEP----------ELPRPRHNPPLYRKDLHLSSALLGGEVDYVMHNILSGKEGQLWE
MIDWV_L         M----EEPEL----------YRP-PRHNPPLYRKDLHLSSALLGGEVDYVIHNILSGKERLLWE
SNVV_L          M----EEPEA------------PPIRHKP-LYRKDLHLSSALLGGEVDYILHNYLAGKRDKLWE
SJCV_L          MYYPGEEPEFGQVDPGEWGEGLQERKAFKPLYRRDLHLSTALLGGEVDYIFHNLLVGNYERLWP
SJCV_P6         M----AGAEP----------------------RIHMESILTHVRSRATDLTRYAIHRLEGHLMR
                *      .*                       * .:.   : : ...  : :  :      *

NYMV_L          DHREVAGIIKSTNIGSYRDLIRLAWRDRRVPSHGDLQLAQAVYTKRLKVALQATNVGLLNWMQR
MIDWV_L         DHKEVAEIIKSNRIGSYRDLIRLAWRDRTVPEDCQLKKYQEIYTKRLRVALKTTNIGLHNWMQR
SNVV_L          DHQLVLRSLQDLDVASYRDFIRNVWG--MTKKTMTQLKYKKDYICHLRISLMALNAGLSNWMQK
SJCV_L          DHKKVADYIKDKDVGSYRDFIREIWALRKPIEDRDYSAAKEEMTTRLRVSLATTNVGLANWTQR
SJCV_P6         HRIEALEVLGPNENSLELRFLQTLLSLPKIRPN----------QADLVVTRTSLINRARRWIDH
                .:  .   :     .    :::                        * ::  :      .* ::

NYMV_L          TSQQGTLQSLIPTLGDDLLNHCLTRELLMLFNQTEENRRQILSVAAQERRNAKVTINGVEFDLI
MIDWV_L         TSKGGTEHSLVPTLGDDLINHCTTRELVMLFLQSEENRRQVLSIAAQEKRRATATINGVHFDFI
SNVV_L          SCIDLKAGTACPTVGEDLINYCCTTDLLNWFEMVVSNKNEIERVAASEIKEDKMTLNGVDLQLI
SJCV_L          TGESAEGGAFCPVLGEDLINHCLEPTIIEWFAHVEANRDEIKRVSAAEAAEAHLQINGVPLQCL
SJCV_P6         PG------------SHSMILYSMSPDVVEWYCHAYSMLIQMKRLVEEKPMDAELSLFGHKVQLK
                .             ...:: :.    ::  :        ::  :   :       : *  .:

NYMV_L          GDLVVYTPSRSDYKIVAPYTALLALTGMTDARFTTLLYARVADYWKKYPETSLYSECIEFFCKA
MIDWV_L         GDLMIYQPHRADYKIVAPYTTLLAVVGMTDARFTTLLYARVADYWKKYPNTSLYQECLAFFCKA
SNVV_L          GDIVILKDKTT--VTPAPYITALAIAGMTNSRFTTLLYARVADYWKKYPSISLYQKCSEFFEAA
SJCV_L          GDIVTLDLKGPSRVIVAPYTALLAVAGMTNARLTSLLYARLADYQFKYPGYSLYQETLAFYRAA
SJCV_P6         GSLLQLEPFRLNVVRLADYKLLAVIAEMTWFRAASLLYARVCDYYKQYGTYSLYQRLVEVYSAY
                *.::            * *    .:. **  * ::*****:.**  :*   ***..   .:

NYMV_L          DHDLEVLGEDLYGVLKCLPSISIGAVLKHTEIKLESSFLETVMEDLPRSS-LATWMSR-PVTSL
MIDWV_L         DYDLEALGEDIYGVLKCLPSLAIGSVLKHTEIKLTSTFLETVKEDLPKSS-LLDWFSR-PLASL
SNVV_L          DYDLSTLGEDLYGVMKCLPSMAIGAVLKHTEMKLESHFLETVTEDLPASR-LRNIFCS-PVLSL
SJCV_L          DEDLKTYGEDIYSILKCMPSMAIGAVLKHTESKINSQFLETVIEDLPAQSRVLQFFTR-DILTL
SJCV_P6         DTDCKFEGELFYKIRDEMSAAMAIATQGWLGPMWYSQSYEEPHPGLH-QTALYRISSREPLCS-
                * * .  ** :* : . :.:    :.         *   *    .*     :

NYMV_L          EAAHVRLEVSGLWKTMGHPFINVSSSVAELRARGTSPAVPTAAEAGEDLACFFKKYWCKVYCKK
MIDWV_L         EEAHVRLELSGLWKTMGHPFINVASSVMELRARGTSPAVPTAEAAGEDLACFFKKYWCRVYCKK
SNVV_L          EEAHVRLELSGLWKTMGHPFIQIESSILELRAKGTSPAVPTAREGGEDLANFFKKYWCHSYFKK
SJCV_L          TEAHIRLELSGLWKTMGHPFIEVLKSVNELRKKGTSPAVDTAAAAGEELAAFFKKYWCRAFCKR
SJCV_P6         KEAQVYRDAVMLVETYGP----------HIRPECDLPCHPWSCAEVDEVLGLKN----------
                  *::  :   * :* *           .:* .   *.   :    :::  : :

NYMV_L          HHCWPPLANPHELPEALRECYLKGTWEEPSPGSWAYDLWKNMEFKPHLDFDYSIDTSELL---
MIDWV_L         HHSWPPIRNPSCLPPFLLECYQKGTWDEPTPGSWAYEHWRDVEFEPHLDFDYSIDTSELL---
SNVV_L          HHRWPPLTGEQSLPLHIREAYKKGTWEEPSVGAWSHNHWKDVSFLPHMDFDYSIDTSELL---
SJCV_L          NHKWPPLVNAQDLPPALLDSYRRGVWDEPAPGAWSHDLFREIELEPHVDFDYSIDTSELL---
SJCV_P6         ----------------------------PSPPPLSQSDWDESDLPPGYDEC
                                            *:  . : . : : .: *  *
```

Suppl. Figure S1. ClustalX amino acid sequence alignment of the SJCV P6 protein and the N-terminal regions of the L proteins of SJCV and other members of the genus *Nyavirus*. Amino acids conserved the between nyavirus L proteins and the SJCV P6 protein are shaded.

Suppl. Table S1. Amino acid sequence homology (p-distances) of the L proteins of SNVV and selected nyamiviruses.

**Genus**	***Nyavirus***				***Orinovirus***	***Socyvirus***	***Berhavirus***			***Crustavirus***			***Tapwovirus***		
**Virus**	SJCV	NYAV	MIDWV	SNVV	OCOV	SbCNV-1	BhRLV-4	BhRLV-5	BhRLV-3	BhRLV-6	WlCV-12	WzCV-1	WzTV-1	FexV-4	FfuV-1
SJCV															
NYAV	63.1														
MIDWV	63.9	82.4													
SNVV	62.4	66.7	67.0												
OCOV	30.4	30.6	31.3	29.8											
SbCNV-1	28.7	29.8	29.7	29.5	26.5										
BhRLV-4	28.3	28.5	28.5	28.0	27.0	26.3									
BhRLV-5	29.5	29.7	30.0	28.9	27.6	25.0	44.6								
BhRLV-3	30.0	31.6	31.1	31.2	27.7	27.2	32.5	33.4							
BhRLV-6	23.9	22.9	23.0	22.8	24.5	23.0	21.0	21.9	23.1						
WlCV-12	25.1	25.8	25.8	24.7	24.7	23.7	24.4	23.0	24.5	33.9					
WzCV-1	24.6	23.8	22.9	23.9	25.6	22.6	22.2	22.2	23.2	47.7	36.5				
WzTV-1	26.3	27.2	26.7	26.1	26.5	26.8	22.8	23.8	26.4	21.3	23.0	22.8			
FexV-4	31.5	31.8	32.2	32.5	42.0	27.5	28.1	27.8	30.9	23.4	25.4	23.6	24.9		
FfuV-1	30.5	31.4	31.8	32.1	42.2	27.7	27.9	27.6	30.2	23.4	25.5	23.7	25.2	86.1	

Suppl. Table S2. Amino acid sequence homology (p-distances) of the PA proteins of MCRV and selected orthomyxoviruses.

	MCRV	LCHV	QRFV	TLKV	ARAV	WFBV	BhOMV1
MCRV							
LCHV	38.6						
QRFV	21.0	21.1					
TLKV	21.9	21.0	75.2				
ARAV	20.5	22.6	52.9	55.1			
WFBV	20.1	21.8	54.5	54.8	54.8		
BhOMV1	21.2	21.9	29.6	29.2	29.2	30.4	

Suppl. Table S3. Amino acid sequence homology (p-distances) of the PB1 proteins of MCRV and selected orthomyxoviruses.

	MCRV	LCHV	QRFV	TLKV	ARAV	WFBV	BhOMV1
MCRV							
LCHV	60.4						
QRFV	40.2	39.8					
TLKV	39.4	39.6	86.9				
ARAV	40.0	39.6	71.4	71.7			
WFBV	39.6	39.8	70.4	70.8	73.4		
BhOMV1	44.7	41.0	43.0	43.6	46.9	45.4	

Suppl. Table S4. Amino acid sequence homology (p-distance) of the PB2 proteins of MCRV and selected orthomyxoviruses.

	MCRV	LCHV	QRFV	TLKV	ARAV	WFBV	BhOMV1
MCRV							
LCHV	32.7						
QRFV	15.9	17.3					
TLKV	15.9	16.7	84.1				
ARAV	16.1	15.4	53.9	53.7			
WFBV	17.0	17.6	53.4	54.3	55.4		
BhOMV1	13.7	14.7	25.7	24.7	25.9	24.6	

Suppl. Table S5. Amino acid sequence homology (p-distances) of the NP proteins of MCRV and selected orthomyxoviruses.

	MCRV	LCHV	QRFV	TLKV	ARAV	WFBV	BhOMV1
MCRV							
LCHV	39.5						
QRFV	21.6	26.0					
TLKV	21.4	26.8	87.9				
ARAV	21.6	25.4	49.7	52.0			
WFBV	21.4	25.4	48.0	49.9	52.4		
BhOMV1	25.6	26.2	31.3	32.6	31.7	30.9	

Suppl. Table S6. Amino acid sequence homology (p-distances) of the HA proteins of MCRV and selected orthomyxoviruses.

	MCRV	LCHV	QRFV	TLKV	ARAV	WFBV	BhOMV1
MCRV							
LCHV	44.0						
QRFV	25.6	22.7					
TLKV	25.2	23.3	85.7				
ARAV	25.0	24.2	43.7	44.3			
WFBV	25.0	23.1	45.4	45.4	48.1		
BhOMV1	23.5	23.9	29.0	27.7	26.7	23.5	

Suppl. Table S7. Amino acid sequence homology (p-distances) of the L proteins of MCRV and selected peribunyaviruses.

Genus		*Shangavirus*	*Herbevirus*			unassigned		*Pacuvirus*			*Orthobunyavirus*																						
**Virus**	**BBAV**	**SgIV-1**	**TAIV**	**HEBV**	**KIBV**	**AKHV**	**KHUV**	**PACV**	**TAPV**	**CAMV**	**BUNV**	**OROV**	**LACV**	**CVV**	**CEV**	**TRIV**	**WYOV**	**KRIV**	**CAPV**	**ALJV**	**BWAV**	**AKAV**	**SBV**	**GMAV**	**CARV**	**SIMV**	**KKV**	**TETEV**	**GROV**	**THIV**	**LEAV**	**ZEGV**	**TATV**
**BBAV**																																	
**SgIV-1**	27.5																																
**TAIV**	29.1	28.4																															
**HEBV**	29.3	28.8	82.3																														
**KIBV**	29.6	28.6	81.6	83.1																													
**AKHV**	31.4	29.5	34.4	33.8	33.5																												
**KHUV**	31.4	29.5	34.4	33.8	33.5	99.8																											
**PACV**	29.0	27.6	29.4	29.4	29.2	34.1	34.1																										
**TAPV**	29.7	27.6	30.3	30.2	30.4	35.0	35.0	72.6																									
**CAMV**	29.7	26.9	29.8	29.5	29.7	35.3	35.3	73.2	79.9																								
**BUNV**	30.6	27.6	30.4	30.5	29.8	33.7	33.7	46.4	47.2	47.1																							
**OROV**	29.1	28.0	29.7	30.1	29.9	33.7	33.7	47.4	48.7	48.2	54.5																						
**LACV**	29.6	28.0	30.8	30.3	30.2	33.3	33.3	47.2	47.6	47.5	59.4	56.0																					
**CVV**	30.5	27.8	30.7	30.2	30.4	33.8	33.8	46.3	47.3	47.4	84.1	54.0	59.0																				
**CEV**	29.6	27.8	31.1	30.5	30.3	33.1	33.1	47.1	47.3	47.4	59.0	55.6	91.1	58.6																			
**TRIV**	30.0	28.0	30.6	29.8	29.6	33.3	33.3	47.4	48.8	48.5	58.9	56.3	83.4	58.6	82.8																		
**WYOV**	30.0	27.2	30.7	30.1	30.2	34.6	34.6	46.8	47.1	47.6	69.9	53.2	58.1	69.8	58.3	58.7																	
**KRIV**	29.9	27.0	31.1	30.9	30.4	34.3	34.3	46.4	46.6	46.4	69.5	53.9	58.1	69.3	57.8	58.7	66.2																
**CAPV**	30.7	28.1	30.1	30.5	30.4	34.5	34.6	46.0	47.8	47.4	54.1	55.4	55.4	53.6	55.3	54.8	53.9	54.1															
**ALJV**	30.5	27.4	29.9	29.9	29.9	32.4	32.4	46.0	45.5	46.0	53.3	51.4	59.0	53.7	58.8	59.6	53.4	52.6	51.3														
**BWAV**	29.7	28.1	29.2	29.3	29.3	33.8	33.8	47.9	47.9	48.6	57.4	55.3	67.1	58.2	66.6	66.8	57.2	56.9	54.0	57.7													
**AKAV**	29.9	27.1	28.9	29.0	29.1	33.1	33.1	46.9	47.1	46.9	53.1	61.9	53.9	52.9	53.9	54.8	52.7	52.3	53.6	51.6	53.8												
**SBV**	29.3	27.3	29.3	29.6	29.2	33.0	33.0	47.1	46.9	47.3	52.4	61.2	53.7	51.9	53.9	54.2	51.4	51.3	53.3	50.6	52.6	73.4											
**GMAV**	30.3	28.4	30.5	30.7	30.4	33.5	33.5	47.1	48.0	47.4	53.8	55.8	55.2	53.2	54.1	55.6	53.3	54.4	82.2	50.9	54.0	53.2	53.8										
**CARV**	30.4	28.4	30.0	30.0	30.3	33.3	33.3	47.1	47.9	47.9	55.4	56.6	55.6	54.9	55.5	55.5	54.5	54.1	72.8	51.7	54.8	54.6	53.8	73.5									
**SIMV**	29.4	27.6	29.4	29.9	29.6	32.7	32.7	46.6	46.5	46.8	52.1	62.2	53.3	52.1	53.3	53.9	51.2	52.1	53.9	50.5	52.5	72.8	71.9	53.6	53.8								
**KKV**	29.9	27.3	30.8	30.2	30.1	34.2	34.2	46.6	46.3	47.0	55.9	55.4	61.6	56.0	60.9	61.9	54.7	57.1	53.7	54.5	59.0	52.9	53.6	54.4	55.2	52.7							
**TETEV**	29.6	28.6	31.7	31.5	31.5	34.6	34.7	48.0	48.5	48.4	55.1	56.5	53.9	55.3	54.0	54.6	53.0	54.3	55.4	51.5	54.4	54.9	54.6	56.0	56.6	55.3	54.6						
**GROV**	29.5	28.1	31.4	31.2	30.4	34.2	34.2	45.9	46.5	45.9	57.6	50.1	53.3	58.9	54.2	55.5	59.5	57.6	50.3	50.4	53.0	49.8	49.0	51.3	51.6	49.4	51.5	51.5					
**THIV**	29.1	27.4	28.8	28.5	28.6	32.7	32.7	46.0	46.4	47.1	54.1	70.1	55.3	54.1	54.8	55.2	53.5	53.8	56.2	51.0	54.3	62.1	62.2	56.4	56.4	64.0	55.0	57.1	49.5				
**LEAV**	30.0	27.2	29.8	28.9	29.4	33.9	33.9	46.8	46.6	46.6	52.6	62.3	54.6	52.0	54.6	54.2	52.7	53.1	54.1	51.0	53.6	57.4	57.4	55.0	55.2	58.7	53.8	54.6	49.1	62.0			
**ZEGV**	30.5	27.7	30.3	30.4	29.9	33.7	33.7	47.2	47.8	48.0	54.4	57.3	55.8	54.2	55.3	56.3	52.4	54.7	67.7	50.6	54.6	54.5	53.3	69.5	69.1	54.2	54.2	56.2	51.4	56.5	55.3		
**TATV**	29.1	27.9	29.4	29.7	30.0	33.2	33.2	45.0	46.1	46.3	54.0	51.8	56.7	53.8	57.1	56.3	53.2	53.3	52.3	52.6	54.7	51.1	50.9	52.4	53.6	49.7	53.8	51.9	51.6	51.2	50.5	51.5	

Suppl. Table S8. Amino acid sequence homology (p-distances) of the N proteins of MCRV and selected peribunyaviruses.

Genus		*Shangavirus*	*Herbevirus*			unassigned		*Pacuvirus*			*Orthobunyavirus*																						
**Virus**	**BBAV**	**SgIV-1**	**TAIV**	**HEBV**	**KIBV**	**AKHV**	**KHUV**	**PACV**	**TAPV**	**CAMV**	**BUNV**	**OROV**	**LACV**	**CVV**	**CEV**	**TRIV**	**WYOV**	**KRIV**	**CAPV**	**ALJV**	**BWAV**	**AKAV**	**SBV**	**GMAV**	**CARV**	**SIMV**	**KKV**	**TETEV**	**GROV**	**THIV**	**LEAV**	**ZEGV**	**TATV**
**BBAV**																																	
**SgIV-1**	16.8																																
**TAIV**	17.8	13.6																															
**HEBV**	19.4	12.6	68.1																														
**KIBV**	20.9	13.6	63.9	74.9																													
**AKHV**	18.8	14.1	16.8	19.4	20.4																												
**KHUV**	18.8	14.1	16.8	19.4	20.4	100																											
**PACV**	20.4	22.0	18.3	17.8	17.3	21.5	21.5																										
**TAPV**	19.9	20.4	19.4	18.8	19.4	18.8	18.8	56.0																									
**CAMV**	20.4	17.8	15.7	16.8	17.8	21.5	21.5	59.7	71.2																								
**BUNV**	18.8	19.9	23.6	24.1	21.5	22.5	22.5	36.6	31.4	27.7																							
**OROV**	16.2	17.3	23.6	22.0	23.0	18.8	18.8	35.6	30.9	31.9	45.5																						
**LACV**	18.8	22.5	20.9	23.0	23.0	22.0	22.0	31.4	27.7	28.8	46.1	47.1																					
**CVV**	18.8	20.9	23.6	24.1	22.5	23.6	23.6	36.1	31.9	28.8	91.1	46.1	47.6																				
**CEV**	18.3	20.4	22.0	22.5	20.9	22.5	22.5	30.4	27.2	28.8	45.5	47.6	88.0	47.1																			
**TRIV**	19.4	22.5	23.6	24.1	24.6	19.9	19.9	29.3	27.2	27.7	47.1	45.5	80.1	47.1	80.0																		
**WYOV**	20.9	22.5	22.0	23.0	23.0	21.5	21.5	34.0	30.9	27.7	63.9	43.5	52.4	63.9	50.8	51.3																	
**KRIV**	19.4	19.9	23.0	22.0	21.5	20.9	20.9	34.6	28.8	28.3	72.8	49.7	51.8	72.3	50.8	49.7	71.7																
**CAPV**	20.4	20.4	20.9	18.8	18.3	23.0	23.0	35.6	29.3	29.8	48.2	41.9	46.6	47.1	47.1	48.7	45.0	48.7															
**ALJV**	22.0	15.7	20.4	18.3	20.4	18.8	18.8	31.9	28.3	25.1	38.7	36.1	37.7	39.3	38.7	37.7	37.7	39.3	39.8														
**BWAV**	18.8	20.9	22.0	21.5	22.0	20.9	20.9	34.6	28.8	29.8	48.2	47.1	70.2	50.3	71.7	71.2	51.8	50.8	47.6	39.8													
**AKAV**	17.8	21.5	22.5	19.4	20.4	19.9	19.9	38.7	31.9	33.0	45.0	70.2	50.3	46.6	50.3	48.7	47.1	46.6	45.5	38.2	51.8												
**SBV**	17.8	19.9	21.5	18.8	22.0	19.9	19.9	38.2	30.9	31.9	44.0	70.7	49.7	44.0	48.2	48.2	46.6	46.1	42.4	36.1	50.8	81.2											
**GMAV**	22.0	16.8	18.8	18.3	19.4	20.4	20.4	33.0	26.7	28.8	45.5	41.4	45.5	45.5	45.0	47.1	45.5	48.2	71.7	38.7	49.7	42.9	43.5										
**CARV**	17.8	16.8	21.5	20.4	19.9	20.4	20.4	31.9	30.4	26.2	47.1	45.0	45.5	46.1	47.1	48.2	43.5	42.4	51.3	40.8	49.2	42.9	42.9	50.8									
**SIMV**	16.8	21.5	23.6	20.9	21.5	19.9	19.9	37.2	32.5	34.0	46.6	75.9	48.7	47.1	48.2	46.6	46.1	47.6	42.9	34.6	47.6	82.7	82.2	42.9	43.5								
**KKV**	17.3	19.4	22.5	24.1	23.6	18.8	18.8	33.0	30.9	30.4	50.3	46.6	62.8	52.4	60.7	64.9	57.6	56.5	50.3	42.4	59.7	45.0	43.5	47.6	45.0	45.0							
**TETEV**	18.3	19.9	18.3	18.8	18.8	17.8	17.8	29.3	28.3	28.3	39.3	35.1	41.4	38.7	39.3	40.8	40.3	39.8	41.9	33.0	42.4	34.6	37.7	40.3	39.8	35.1	39.3						
**GROV**	18.3	22.0	20.9	22.5	19.9	24.1	24.1	33.5	28.3	26.7	69.6	44.0	50.3	70.7	49.7	49.7	73.8	73.3	47.6	36.1	51.3	46.1	45.0	45.5	44.0	45.5	52.9	41.9					
**THIV**	18.8	18.3	22.5	21.5	20.9	18.8	18.8	36.6	29.3	30.4	46.6	67.5	47.6	46.6	49.2	48.2	43.5	44.0	39.8	38.7	50.8	62.8	62.3	42.4	44.5	63.9	45.5	35.6	47.6				
**LEAV**	19.4	21.5	22.0	23.6	24.1	20.4	20.4	35.6	35.1	36.1	44.5	53.9	48.7	45.0	46.1	47.6	44.0	44.5	42.4	39.8	49.7	56.0	55.5	41.9	44.5	53.4	45.5	34.0	42.9	56.5			
**ZEGV**	18.8	19.9	19.4	20.9	21.5	22.0	22.0	33.0	29.8	29.3	45.5	42.4	45.5	46.1	44.5	45.0	45.0	46.6	48.2	37.7	48.7	44.0	42.4	47.1	47.1	41.9	46.1	38.7	45.0	45.0	39.8		
**TATV**	23.6	17.8	20.9	22.5	22.5	22.0	22.0	27.2	28.8	26.7	38.7	37.7	42.9	37.7	44.0	40.8	42.4	38.2	47.1	41.4	44.0	39.3	38.2	42.4	44.0	36.6	41.9	35.1	40.3	37.7	40.8	40.8	
